# NAC Transcription Factors as Positive or Negative Regulators during Ongoing Battle between Pathogens and Our Food Crops

**DOI:** 10.3390/ijms22010081

**Published:** 2020-12-23

**Authors:** Zhiyuan Bian, Huanhuan Gao, Chongying Wang

**Affiliations:** Ministry of Education Key Laboratory of Cell Activities and Stress Adaptations, School of Life Sciences, Lanzhou University, Lanzhou 730000, China; bianzhy14@lzu.edu.cn (Z.B.); gaohh@lzu.edu.cn (H.G.)

**Keywords:** NAC TFs, pathogens, food crops, phytohormones, reactive oxygen species

## Abstract

The NAC (NAM, ATAF1/2, and CUC2) family of proteins is one of the largest plant-specific transcription factor (TF) families and its members play varied roles in plant growth, development, and stress responses. In recent years, NAC TFs have been demonstrated to participate in crop-pathogen interactions, as positive or negative regulators of the downstream defense-related genes. NAC TFs link signaling pathways between plant hormones, including salicylic acid (SA), jasmonic acid (JA), ethylene (ET), and abscisic acid (ABA), or other signals, such as reactive oxygen species (ROS), to regulate the resistance against pathogens. Remarkably, NAC TFs can also contribute to hypersensitive response and stomatal immunity or can be hijacked as virulence targets of pathogen effectors. Here, we review recent progress in understanding the structure, biological functions and signaling networks of NAC TFs in response to pathogens in several main food crops, such as rice, wheat, barley, and tomato, and explore the directions needed to further elucidate the function and mechanisms of these key signaling molecules.

## 1. Introduction

Crops are constantly challenged by a variety of abiotic and biotic factors that have negative impacts on their growth, development and yields [1]. Biotic stressors, mainly bacteria, fungi and viruses, can cause diseases and impact agricultural crops, forestry plantations, and native plant communities [2]. To resist or tolerate these adverse external stresses, plants have evolved many effective stress response strategies, including changes in morphology, establishing defensive systems, and constructing physiological, biochemical and molecular regulatory networks.

Plants have two main branches of defense against various pathogens: PAMP (pathogen-associated molecular patterns)-triggered immunity (PTI) and effector-triggered immunity (ETI) [3]. PTI is a basal defense mechanism in plants that is triggered by the recognition of PAMPs through PRRs (pattern recognition receptors) at the plant cell surface [4]. ETI is an accelerated and amplified PTI response, which arises from the interactions between plant resistance proteins and pathogen effector proteins [5,6]. Upon being attacked by pathogens, plants will activate a series of complex molecular regulatory networks, such as reactive oxygen species (ROS) signaling [7], phytohormone signaling [8], changes in redox status [9], and inorganic ion fluxes [10], to prevent further pathogen invasion. 

Much of the plant response to pathogens involves transcriptional reprogramming. Plants have established high-efficiency gene expression networks to regulate multiple specific stress responsive genes in a coordinated manner. Such regulation of the large-scale expression of genes requires a concerted function of different types of transcription factors (TFs). TFs specifically bind to cis-elements and/or trans-acting factors in the promoters of target genes and act as transcriptional activators or repressors. Several TF families, such as NAC (NAM, ATAF and CUC), MYB (myeloblastosis-related proteins), WRKY (WRKYGQK), bZIP (basic leucine zipper domain), bHLH (basic helixloop-helix), CAMTA (CaM-binding transcription activator) and ERF/AP2 (ethylene responsive factor/apetala2), have crucial roles in abiotic and biotic stress responses [11]. So far, a large number of studies have proved that NAC TFs play roles in plant growth, development, abiotic stress response, and disease resistance [11,12,13,14,15,16,17]. 

Pathogens directly affect plant growth and development and decrease the quality and yields of the crop. Three major cereal crops, namely rice (*Oryza sativa*), maize (*Zea mays*), and wheat (*Triticum aestivum*), provide two-thirds of the food consumed all over the world [18]. Barley (*Hordeum vulgare*) and soybean (*Glycine max*) are also important crops, in the grain and legume families, while tomato (*Solanum lycopersicum*) is a fruit crop. NAC TFs comprise a gene family with 151 members in rice [19,20,21], 157 in maize [22], 559 in wheat [23], 167 in barley [24], 152 in soybean [25], and 93 in tomato [26]. Recent studies demonstrate that NAC TFs can be induced by pathogen infection, regulate the expression of downstream genes, and integrate hormone and other signals to offer plants resistance against pathogens. In this review, we introduce the systematic classification and structural characteristics of NAC TFs, focus on recent progress in defining their biological functions and signal regulation networks in disease response of several main food crops (including rice, wheat, barley, tomato), and discuss future research directions needed to understand how NAC TFs promote the resistance against pathogens.

## 2. Overview of NAC TFs

NAC TFs constitute one of the largest groups of plant-specific TFs, with more than 100 members in most plants, including model plants like Arabidopsis or most crops, and the highest reported number of 559 in wheat [23]. The NAC acronym is derived from three reported proteins that contain a highly conserved domain in their N-terminal region (the NAC domain): (i) NAM (No Apical Meristem), (ii) ATAF1/2 (*Arabidopsis thaliana* Transcription Activator Factor 1/2) and (iii) CUC2 (Cup-shaped Cotyledon 2) [20,21]. NAC TFs play essential roles in diverse biological processes, such as growth, development, senescence and morphogenesis, and are widely involved in signaling pathways in response to different phytohormones and multiple abiotic and biotic stress [27,28,29].

### 2.1. Phylogeny and Classification of the NAC TFs

NAC proteins are widespread in land plants, from “simple” bryophytes to “complex” angiosperms, and also in freshwater green algae (Charophytes) [30,31,32], a sister clade to land plants and likely their evolutionary root [33]. NAC proteins originated about 725–1200 million years ago (Mya), during the diversification of the charophytes/embryophytes (the Streptophytes), experienced a first expansion in bryophytes about 470 Mya, and a second expansion in angiosperms during the early Cretaceous period [27]. Along with the divergence of vascular plants, NAC proteins extensively expanded [30] shortly before the origination and radiation of angiosperms through different (segmental or tandem) duplications [31,34,35]. In angiosperms, there are two detectable evolutionary pathways of the NAC proteins: ancient duplications that occurred before the divergence of dicots and monocots and recent duplications in a particular lineage of dicots or monocots [30,36]. As a result of duplication, some family members possess redundant functions [37,38].

According to the clustering analyses by Zhu et al., NAC proteins consist of 21 subfamilies among bryophytes and vascular plants [30]. Among these subfamilies, 15 were found only in angiosperms, while the other six occur in both flowering plants and earlier diverged lycophytes [30,33]. In 2015, another team recategorized NAC proteins into six major orthologous groups (Group I-VI) using more than 2000 non-redundant sequences from 24 different species of green plants by comparative genomic and gene functional analyses [31]. NAC group I is considered the basal NAC group, because its members are involved in secondary wall and wood formation, which might have been essential to water conduction or support during the adaptation of plants to land environments [39,40]. Group II NACs function in specific developmental processes among different organs, as well as other processes such as ethylene-auxin pathways. NAC group III is named the TMM (transmembrane motifs) Group, as 142 of the 164 NAC proteins in this group contained a TMM in their C-terminal regions that can anchor them in biomembranes (especially on the endoplasmic reticulum), and can be hydrolyzed by proteolytic enzymes activated by signaling [41,42,43]. The members in NAC group IV have diverse functions, such as ANAC009, which controls the reorientation and timing of cell division, and ANAC042 which regulates longevity of Arabidopsis. NAC group V is proposed to be the Stress Group, as most of its members are involved in stress responses. Finally, NAC group VI contains many species-specific sequences, which experienced whole-genome duplication events through their evolutionary history [31].

### 2.2. Structure and Function of NAC Proteins

NAC proteins usually contain two relatively independent domains: one well-conserved N-terminal NAC domain of 151–159 amino acids and a relatively divergent C-terminal Transcriptional Activation Region (TAR) [20,44,45]. There are a few kinds of atypical NAC proteins, which have variations in either their NAC domains or TAR [46]. The NAC domain exists in all NAC proteins, and the most conserved consensus sequences are D-D/E-L-I/V, E-W-Y-F-F, G-Y-W-K, and M-H-E-Y [46]. The NAC domain contains nuclear localization signals (NLSs) and its main function is translocating the protein from the cytoplasmic matrix to the nucleus [44,47] and forming homo- or heterodimers, that the state in which these proteins bind DNA [48,49,50]. Some proteins also contain nuclear export signals (NESs) in the NAC domain, which might mediate their export out of the nucleus for degradation after their missions are completed [32]. Crystallographic structures of ANAC019 from Arabidopsis and SNAC1 (STRESS RESPONSIVE NAC1) from rice revealed that the NAC domain contains mainly twisted antiparallel β-sheet(s) flanked by a few α-helices. The central β-sheet is responsible for dimerization of the proteins, by forming stabilized salt bridges between conserved amino acids in two subunits, and also for interacting with the major groove of DNA in the dimer form [49,50,51]. The NAC domain can be further divided into five subdomains, designated A-E, distinguished by blocks of heterogeneous amino acids or gaps, among which subdomains A, C, and D are highly conserved compared to B and E. It is assumed that subdomain A promotes functional dimerization, subdomains C and D play parts in DNA binding, due to their content of basic amino acids, and in nuclear translocation of the protein with the help of the NLSs inside the subdomains, while the variable subdomains B and E diversify the roles of the NAC proteins [44,48].

The TAR region, also known as TRR (transcriptional regulatory region), is related to transcriptional regulation and corresponds to the distinct functions of NAC proteins [46,48], acting as both activator and/or repressor to downstream target genes together with the NAC domain [27]. Bioinformatic analysis revealed common motifs in the C-terminal regions of some NAC subfamilies [48,52]. In addition, an α-helical TMM could be found at the C-terminal end of many NAC proteins [43] that contain a conserved N-terminal NAC domain, a variable middle TAR region, and a C-terminal TMM motif [42]. With the help of the TMM, the nascent NAC protein can be anchored to the endoplasmic reticulum membrane or plasma membrane, retaining it outside of the nucleus in a dormant state. Subsequently, the NAC TF can be cleaved through either protease- or ubiquitin proteasome-mediated proteolytic events upon stimulation by internal and/or environmental signals, which would activate the mature nuclear-associated form to enter the nucleus and regulate downstream genes, which is an adaptive strategy to environment [41,42,43]. Recently, transmembrane domains were found in the N-terminal ends of a small number of NAC proteins [32].

## 3. NAC TFs Have Positive or Negative Roles in Crop Disease Resistance

In both model and crop plants, numerous *NAC* genes are induced in response to pathogen infection [46]. Over-expression or silencing of certain *NAC* genes results in enhanced or reduced resistance to pathogens [53,54,55,56], suggesting that NAC TFs could positively or negatively regulate plant defense response. Here, we introduce the roles of NAC TFs during the response to pathogens in several important crops, including rice, wheat, barley, potato, soybean, maize, tomato and lettuce.

### 3.1. Roles of NAC TFs in Rice

Rice is one of the most important food crops in the world, as it is consumed by more than 3 billion people [57]. Rice blast caused by the fungal pathogens *Magnaporthe oryzae* and *M. grisea* is one of the most devastating diseases threatening rice production. Several NACs have been found to respond to rice blast, therefore, it is important to understand the role of these TFs in response to both this disease and other pathogens in rice.

The rice genome is predicted to contain 151 *NAC* genes [19], but only a few have been characterized. OsNAC6 was the first NAC TF found to be involved in disease resistance in rice [58]. *OsNAC6* can be induced by both abiotic and biotic stress, including blast disease. Transgenic rice plants over-expressing *OsNAC6* had improved tolerance to rice blast, dehydration and high salt stress, but exhibited negative effects on growth and yields [58]. Similarly, resistance was obtained from the over-expression of *OsNAC111* or *OsNAC58*. Over-expression of *OsNAC111* or *OsNAC58* in rice increased resistance to *M. oryzae* or the bacterial blight pathogen *Xoo* (*Xanthomonas oryzae* pv. *oryzae*), respectively [59,60]. In addition, inoculation with *M. oryzae* can induce *OsNAC111* expression, which then activates several downstream defense genes, such as *PR1* (*PATHOGENESIS-RELATED1*) and *PR8*, suggesting that the OsNAC111 TF positively regulates the expression of a specific set of *PR* genes in the rice blast response [59]. ONAC122 and ONAC131 also play positive regulatory roles during rice blast resistance and can be induced by infection with *M. grisea* and treatment with exogenous defense signaling molecules, such as salicylic acid (SA), methyl jasmonate (MeJA), or 1-aminocyclopropane-1-carboxylic acid (a precursor of ethylene) [61]. Metabolomics analysis was recently used, for the first time, to explore the molecular mechanism of NAC-mediated disease resistance. Liu et al. found that ONAC066 promotes the resistance against fungal blast and bacterial blight in rice by regulating the accumulation of soluble sugars and amino acids as well as the up-regulation of the *PR* gene [62].

The hypersensitive response (HR) of plants against disease is a type of programmed cell death. Kaneda et al. found that over-expression of *OsNAC4* in rice could lead to HR cell death, accompanied with the loss of plasma membrane integrity, fragmentation of nuclear DNA, and typical morphological changes. HR cell death is noticeably decreased in *OsNAC4* knock-down lines after induction by an avirulent pathogen N1141 [63], suggesting that OsNAC4 is a key positive regulator of plant hypersensitive cell death. In addition, the *osnac60* mutant had increased susceptibility to *M. oryzae*, while the over-expressing plant had an enhanced defense response, including increased programmed cell death, ROS accumulation, and callous deposition and up-regulation of defense-related genes [64].

On the contrary, Yoshii et al. found that mutation of *RICE DWARF VIRUS MULTIPLCATION 1* (*RIM1*), which encodes a NAC TF, resulted in a loss of susceptibility to Rice Dwarf Virus (RDV) but not to Rice Transitory Yellowing Virus (RTYV) and Rice Stripe Virus (RSV) [65]. The accumulation of virus capsid protein was significantly reduced in *rim1* mutant plants inoculated with RDV, which impairs the multiplication of the virus, while proliferation of the virus was stimulated with over-expression of *RIM1*. Therefore, it was proposed that RIM1 negatively regulates rice resistance to RDV, by acting as a host factor that is required for multiplication of the virus [65].

This summary shows that 9 NAC TFs (OsNAC4, OsNAC6, OsNAC58, OsNAC60, ONAC066, OsNAC111, ONAC122, ONAC131, and RIM1) have been validated to take part in defense responses against pathogen attack. OsNAC6, OsNAC58, OsNAC60, ONAC066, OsNAC111, ONAC122 and ONAC131 positively regulate rice resistance to fungus *Magnaporthe*, while RIM1 negatively regulates rice resistance to the virus RDV.

### 3.2. Roles of NAC TFs in Wheat

Wheat is the most widely distributed food crop and occupies the largest planting area in the world [66]. The planting area and total output account for one third of all food crops, and about one-third of the population of the world uses wheat as its main food [67]. Thus, it is quite urgent to develop novel wheat varieties that have improved yield potential and increased tolerances to biotic and abiotic stresses. There are 559 *TaNAC* genes in the wheat genome, 186 of which were recently identified during infection of the resistant hexaploid wheat line N9134 with the fungal agents that cause rust and powdery mildew [23].

Powdery mildew, caused by the biotrophic *Blumeria graminis f.* sp. *tritici* (*Bgt*), is a widespread disease in wheat [68]. Zhou et al. isolated and characterized three wheat TaNAC6s, TaNAC6-A, TaNAC6-B and TaNAC6-D. Analysis of the expression patterns of the TaNAC6s showed that *TaNAC6-A* and *TaNAC6-D* were up-regulated early after *Bgt* inoculation. Over-expression of the *TaNAC6s* enhanced the resistance to *Bgt*, while silencing them reduced the resistance in wheat, indicating that the TaNAC6s play positive roles in resistance against *Bgt* [69]. Recently, wheat TaNACL-D1 was demonstrated to be involved in the disease response to Fusarium head blight (FHB) [70], a devastating disease of wheat and barley in humid and semi-humid regions of the world. TaNACL-D1 was recently shown to interact with TRITICUM AESTIVUM FUSARIUM RESISTANCE ORPHAN GENE (TaFROG), which enhances wheat resistance against FHB, and lines over-expressing *TaNACL-D1* were more resistant to FHB disease [70,71].

Previous studies have shown that wheat *TaNAC4* and *TaNAC8* shared high homology with rice *OsNAC4* and *OsNAC8*. *TaNAC4* and *TaNAC8* expression is induced by infection with *Puccinia striiformis f.* sp. *tritici* (*Pst*, which causes stripe rust) as well as by treatments with MeJA or ethylene (ET), but not by treatments with abscisic acid (ABA) or SA [72,73]. This indicates that TaNAC4 and TaNAC8 of wheat might be two important components in defense-signaling pathway and play essential roles in resistance to pathogen.

*TaNAC1* is strongly expressed in wheat roots and is involved in responses to *Pst* infection and treatments with defense-related hormones. Knockdown of *TaNAC1* enhances resistance to *Puccinia* stripe rust in wheat, while over-expression of *TaNAC1* in Arabidopsis enhances susceptibility and reduces systemic-acquired resistance to *Pseudomonas syringae* pv *tomato* DC3000 (*Pst* DC3000). Similarly, knockdown of *TaNAC21/22* in wheat enhanced resistance against *Puccinia* stripe rust [74,75]. Silencing of *TaNAC2* or *TaNAC30* enhances the resistance against stripe rust by significantly increasing H_2_O_2_ generation and decreasing hyphal growth at the early stage of the interaction between *Pst* and wheat [76,77]. These data indicate that TaNAC1, TaNAC2, TaNAC21/22, and TaNAC30 negatively regulate stripe rust resistance in wheat.

### 3.3. Roles of NAC TFs in Barley

Barley is the fourth most important grain crop in the world [78] and, like the other cereal crops, is threatened by various pests and diseases [79]. A search of the public barley sequence database identified 48 *NAC* genes (*HvNACs*), while the expression profiles of 46 *HvNACs* were investigated in various tissues with and without ABA or MeJA treatment. The HvNAC proteins have conserved functions in secondary cell wall biosynthesis, leaf senescence, root development, seed development and hormone-regulated stress response [80].

Barley *HvNAC6* has a high similarity to the rice *OsNAC6* in pathogen resistance. Transient over-expression of the gene in barley increased the penetration resistance of epidermal cells to the powdery mildew pathogen *Blumeria graminis f.* sp. *hordei* (*Bgh*), while silencing of the gene, using RNA interference (RNAi), enhanced the sensitivity to *Bgh*. Silencing of *HvNAC6* also changed the accumulation of ABA, which was not affected by *Bgh* inoculation, indicating that HvNAC6 acts as an ABA-mediated defense response regulator to maintain basal resistance against *Bgh* [81,82]. In Arabidopsis, the expression of the *HvNAC6* homologue *ATAF1* was induced by *Bgh*, and the *ataf1-1* mutant line displayed reduced penetration resistance to *Bgh*. HvNAC6 and ATAF1 play conserved positive roles in penetration resistance in both monocots and dicots, respectively [82].

Another NAC TF in barley is HvSNAC1, which promotes resistance to barley ramularia leaf spot (RLS) disease [83]. RLS is a new emerged barley disease, caused by the ascomycete fungus *Ramularia collo-cygni* and which broke out in Europe a decade ago [84]. Over-expression of HvSNAC1 in barley significantly reduced the severity of RLS, but had no effects on other pathogenic diseases, such as eyespot, powdery mildew, or blast. Further analysis showed that dark-induced leaf senescence is delayed in HvSNAC1 over-expression lines, indicating that HvSNAC1 may inhibit plant senescence [83].

### 3.4. Roles of NAC TFs in Tomato and Potato

Tomato is the highest value vegetable and fruit crop worldwide, at an annual production of 100 million tons, and makes a huge nutritional contribution to the human diet [85,86,87]. At the same time, tomato is constantly attacked by various pathogens, causing huge losses in production [88,89,90]. So far, 93 putative NAC proteins were identified in tomato [26]. Similar to other crops, tomato NAC TFs play roles in abiotic and biotic stress responses, as well as the development of the plant [91,92,93,94,95].

Infection by pathogens induces the expression of *SlNAC1* in tomato, but plays dual functions in resistance to different pathogens. *SlNAC1* expression is specifically induced in tomato by the replication enhancer (REn) of Tomato leaf curl virus (TLCV) [96], and its over-expression increases the accumulation of viral DNA in infected cells, indicating that SlNAC1 play negative roles in resistance against TLCV. SlNAC1 is also induced by *Pseudomonas* infection, but plays reversed roles in defense signaling [56]. In *Pseudomonas*-infected plants, expression of *SlNAC1* increased rapidly while degradation of the SlNAC1 protein was suppressed. Further research proved that SlNAC1 could be ubiquitinated by SINA3, a ubiquitin ligase, but the expression of SINA3 was decreased in infected plants. Thus, pathogen infection counteracts the degradation of the SlNAC1 protein. These data suggest that SlNAC1 plays a positive role in resistance to *Pseudomonas* infection [56,97].

The NAC protein, *Solanum lycopersicum* Stress-related NAC1 (SlSRN1), was identified in tomato by virus-induced gene silencing technology [98]. The expression of *SlSRN1* can be significantly induced by infection with *Botrytis cinerea* and *Pst* DC3000, while silencing of *SlSRN1* leads to increased severity of the diseases. Silencing of *SlSRN1* accelerates accumulation of ROS but reduces expression of defense genes after infection by *B. cinerea*. These results demonstrate that *SlSRN1* is a positive regulator of the defense response against *B. cinerea* and *Pst* DC3000 in tomato [98]. Recently, six NAC TFs (SlNAC24, SlNAC20, SlNAC39, SlNAC47, SlNAC61 and SlNAC69) were studied in response to Tomato yellow leaf curl virus (TYLCV) infection in tomato. Four *NAC* genes (*SlNAC20*, *SlNAC24*, *SlNAC47*, and *SlNAC61*) were induced after TYLCV infection in resistant plants, and SlNAC61 played positive roles in response to TYLCV infection, according to Virus-induced gene silencing analysis. Furthermore, the six NAC TFs could interact with protein phosphatase 2C (PP2C), mitogen-activated protein kinase 3 (MPK3), and some defense response TFs, such as WRKY, MYB, and even NAC, by binding the promoters of these genes, indicating that NAC TFs have an complex response mechanism during TYLCV infection [94]. Recently, it was found that SlNAC082, a ribosomal stress mediator, was involved in the process of infection by citrus exocortis viroid (CEVd) in tomato. A higher expression level of *SlNAC082* was detected in the CEVd-infected tomato leaves. CEVd and its derived viroid small RNAs were found to co-sediment with tomato ribosomes in vivo and caused alterations in ribosome biogenesis in the infected tomato plants. The alterations in both the rRNA processing and the induction of *SlNAC082* were correlated with the degree of viroid symptomology [95].

Stomata play an active part in the plant innate immune response, and serve as an entrance for pathogen into plant cells [99]. The genes *JA2* (*Jasmonic Acid 2*) and *JA2L* (*JA2-like*) both encode two NAC TFs that are closely related to ANAC019/ANAC055/ANAC072. These *NACs* were preferentially expressed in guard cells of tomato leaves [100]. In JA2-SRDX (SUPERMAN REPRESSION DOMAIN X) plants, *Pst* DC3000-induced stomatal closure was impaired at 1 h post infection (hpi), and pathogen-triggered stomatal reopening remained normal at 4 hpi, indicating that JA2 is required for *Pst* DC3000-induced stomatal closure but not stomatal reopening. By contrast, the *Pst* DC3000-induced stomatal closure was largely normal at 1 hpi, but the pathogen-triggered stomatal reopening was substantially impaired at 4 hpi in JA2L-AS plants expressing an antisense version of the JA2L cDNA, indicating that JA2L is required for pathogen-regulated stomatal reopening [100].

Potato is one of the four major food crops around the world. A NAC TF, StNACb4 from potato, was identified and characterised. StNACb4 has been shown to promote resistance to bacterial wilt caused by *Ralstonia solanacearum* [101]. Transgenic tobacco plants were generated in which the expression of *StNACb4* was constitutively up-regulated or suppressed using RNAi. StNACb4 was found specifically in the phloem of the vascular system of the stems and leaves, and up-regulated upon infection with *R. solanacearum* or by treatment with SA, ABA and MeJA in transgenic tobacco. Silencing *StNACb4* reduced the tolerance of tobacco to *R. solanacearum*, and over-expression of the gene enhanced the tolerance to this pathogen [101]. These results are consistent with findings on StNAC43, another potato NAC TF, which can increase the deposition of resistance-related metabolites to reinforce the secondary cell wall and improve resistance to late blight disease [102]. These data demonstrate that both StNACb4 and StNAC43 are positive regulators of disease resistance of potato.

### 3.5. Roles of NAC TFs in Other Crops

Maize is not only an important and widely distributed cereal crop, but also a model plant for genetic research [103]. Plant diseases induced by pathogens cause huge yield losses, up to 41.1% every year [104]. Lu et al. identified 157 non-redundant maize *NAC* genes, which were unevenly distributed on 10 maize chromosomes [22]. Further sequence and evolutionary relationship analysis showed that 19 maize *NAC* genes were related to stress responses [105]. *ZmNAC41* and *ZmNAC100* were transcriptionally induced during infection by *Colletotrichum graminicola* and defense signals, and were also expressed during leaf senescence in maize. In addition, *ZmNAC41* was up-regulated in response to the fungal biotroph *Ustilago maydis*. Interestingly, the transcripts of *ZmNAC41* and *ZmNAC100* are induced by JA and SA, respectively, suggesting that *ZmNAC41* and *ZmNAC100* could function in the defense response [106]. When the upstream promoters of maize *NAC* genes were analyzed, a MYC binding site was detected in *ZmNAC15*, *ZmNAC38* and *ZmNAC41*, while a WRKY-binding motif was detected in *ZmNAC15*, *ZmNAC36*, *ZmNAC41,* and *ZmNAC100*. In short, the *ZmNAC15*, *ZmNAC36*, *ZmNAC38*, *ZmNAC41*, and *ZmNAC100* genes all contained potential binding elements for TFs known to be involved in the plant defense network [106].

Soybean is a main source of high-quality proteins and a vegetable oil that provide nutrition for animals and humans [107]. NAC TFs are believed to play vital roles in soybean development and disease resistance. Six NAC-like genes, designated *GmNAC1*–*GmNAC6*, were cloned and characterized from soybean a decade ago. These genes had similar genomic organization and high sequence similarity, especially in the NAC domains, but exhibited different expression patterns during seed development [108]. Subsequently, more NAC proteins were identified in soybean [109], most of which are involved in development and abiotic stress, such as GmNAC30 [110], GmNAC81 [110,111], GmNAC109 [112] and GmNAC8 [113], while up to now only GmNAC42 is reported to be involved in plant disease resistance [114,115,116]. Soybean GmNAC42-1 is a homolog of the Arabidopsis ANAC042-1, which is an indole alkaloid plant antitoxin regulator. Over-expression of *GmNAC42-1* in elicited hairy roots significantly increases the amount of glyceollin in soybean, suggesting this protein is an essential and positive regulator of glyceollin biosynthesis. *GmNAC42* is annotated as a systemic acquired resistance (SAR) gene and functions in soybean disease resistance because glyceollins are defensive metabolites (phytoalexins) derived from isoflavones in soybean [114]. MYB TFs are also involved in the glyceollin gene regulatory network. *GmMYB29A1* and *GmMYB29A2* were up-regulated in hairy roots treated with a wall glucan elicitor from *P. sojae*, and the expression of *GmNAC42-1* and *GmMYB29A1* were increased with the over-expression of *GmMYB29A2*, indicating that *GmNAC42-1* was also regulated by GmMYB29A2 during glyceollin biosynthesis [116].

In lettuce, LsNAC069, a NAC TF with a C-terminal TMM motif, is a target of the RxLR-like effectors of the fungus *Bremia lactucae* [117]. RxLR effectors are characterized by a conserved RxLR (Arg-x-Leu-Arg) motif in the N-terminal domain, and *B. lactucae* secretes potential RxLR effectors during the infection process. *LsNAC069* silencing increases resistance to *Pseudomonas cichorii* bacteria. LsNAC069 is relocalized from the ER to the nucleus when wild-type plants are treated with *Phytophthora capsici* culture filtrate, but this process could be prevented by the protease inhibitor TPCK (N-tosyl-L-phenylalanine chloromethyl ketone), indicating that the LsNAC069 needs proteolytic cleavage to be untethered from the ER and relocalized to the nucleus. However, the susceptibility to *B. lactucae* was not significantly altered in *LsNAC069* silenced lettuce lines, and the process of LsNAC069 relocalization was inhibited upon the expression of *B. lactucae* effectors. Moreover, both co-localization and yeast two-hybrid experiments demonstrated that LsNAC069 could interact with *B. lactucae* effectors. Together these data demonstrate that *B. lactucae* can cause disease in lettuce through its RxLR effectors inhibiting the hydrolysis and relocalization of LsNAC069 from the ER to the nucleus, which suppresses the activation of genes downstream of LsNAC069 [117].

**Figure 1:**
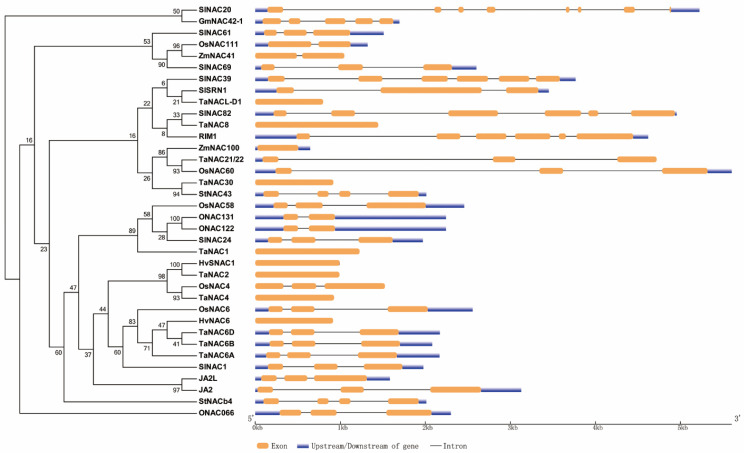
Phylogenetic and gene structure analysis of NAC TFs mentioned in this review. The left is phylogenetic analysis of some NAC TFs from rice, wheat, barley, maize, soybean, tomato and potato. The right is gene maps of the same NAC TFs, showing the numbers of exon and intron, the absence or presence of upstream/downstream sequences and their relative length.

**Figure 2:**
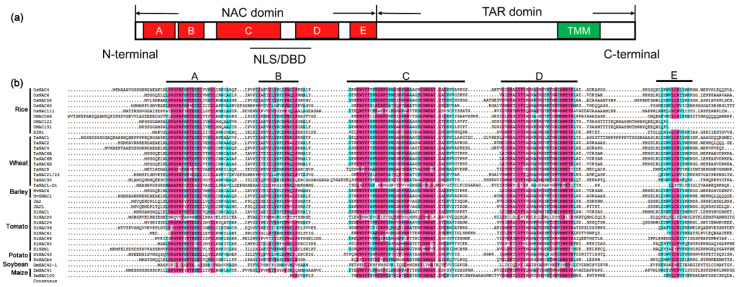
Diagram of the NAC proteins structure and sequence alignment of NAC domains. (**a**) Schematic representation showing a typical NAC protein with a highly conserved NAC domain at the N-terminal which is further divided into five conserved subdomains (**A**–**E**). The NAC domain contains nuclear localization signals (NLSs) and DNA-binding domain (DBD). The C-terminal region is a relatively divergent transcriptional activation region (TAR). In some cases, the C-terminal may have a transmembrane motif (TMM). (**b**) Sequence alignment of NAC domains for most of crops cited in the review. Subdomains A to E are shown by horizontal lines above the sequence. The red color represents amino acids with more than 75% common consensus sequences, and the cyan color represents amino acids with more than 50% common consensus sequences.

As mentioned above, we constructed a phylogenetic tree of NAC TFs cited in the review except LsNAC069 in lettuce, which cannot be searched out, and constructed a diagram of those genes (Figure 1). Meanwhile, we also made a diagram of the NAC domain structure of most NAC protein sequences mentioned here (Figure 2 and Supplementary Table S1).

## 4. Cross-Talk between NAC TFs and Plant Hormones and Signaling Molecules

A plant can sense signals from pathogens during their attack and activate a complicated and finely tuned network composed of reactive oxygen species (ROS) and phytohormone-mediated signaling pathways [118,119,120]. Previous studies have shown that some NAC proteins are involved in modulating these immune signaling pathways.

### 4.1. Cross-Talk between NAC TFs and Phytohormones 

Phytohormones are usually divided into two categories, according to their physiological effects: the first is related to plant growth and development and includes auxin, gibberellins, brassinosteroids and ABA, and the other is defense-related hormones, such as SA, JA and ET [121]. At the same time, these functions are not exclusive, as plant defenses can affect ABA responses and ABA signaling also plays an important role in plant disease resistance [122,123].

#### 4.1.1. Cross-Talk between NAC TFs and SA

SA is a critical signaling molecule that activates defense responses during many plant-pathogen interactions, especially against biotrophs and hemi-biotrophs [124,125,126]. There are two SA biosynthesis pathways, via ISOCHORISMATE SYNTHASE (ICS) and PHENYLALANINE AMMONIA LYASE (PAL), with both starting from chorismate [127,128]. The main SA synthesis route, through the ICS pathway, occurs in the chloroplast and accounts for about 90% of SA production [129]. Most of the produced SA in a plant can be converted into SA O-β-glucoside (SAG) by SA GLUCOSYLTRANSFERASE (SAGT), which is induced by pathogens [130]. In the Arabidopsis NAC triple mutant *anac019anac055anac072*, the basal transcriptional level of *ICS1* was higher and the level of *SAGT* was lower than in wild-type plants. In addition, chromatin immunoprecipitation (ChIP) experiments showed that the DNA samples containing NAC core-binding sites in the *ICS1* and *SAGT1* promoters were precipitated and enriched by ANAC019. Therefore, ANAC019/ANAC055/ANAC072 may act as negative transcriptional regulators of SA accumulation through decreasing SA synthesis and increasing SA metabolism in Arabidopsis by inhibiting ICS and inducing SAGT, respectively [131].

In rice, the SA-mediated signaling pathway is also crucial in activating the innate immune response [132]. In rice treated with SA, two pathogen-responsive NAC TFs, ONAC122 and ONAC131, were strongly induced. Although the two proteins are highly homologous, the expression level of *ONAC131* increased by more than 3 fold 24-48 h post treatment, while *ONAC122* increased only at 48 h after 150 μM SA treatment. These two NAC TFs in rice are both responsive to SA, and their responses are variable [61].

#### 4.1.2. Cross-Talk between NAC TFs and JA/ET

JA and ET are two other defense signaling molecules that regulate the immunity of plants to necrotic pathogens and herbivorous insects [118,133]. The NAC TF from rice, RIM1, is involved in the propagation of RDV and JA signaling [134]. The expression of key enzymes of JA biosynthesis, LIPOXYGENASE (LOX), ALLENE OXIDE SYNTHASE (AOS2) and OPDA REDUCTASE7 (OPR7), were up-regulated in *rim1* mutants, while JA biosynthesis was partially repressed in *RIM1* over-expressed lines, indicating that RIM1 may be a negative regulator of JA signaling in rice [134]. In wheat, TaNAC1 is also a negative regulator of stripe rust resistance. Over-expression of *TaNAC1* in Arabidopsis constitutively induces the expression of *PLANT DEFENSIN 1.2* (*PDF1.2*) and *OCTADECANOID-RESPONSIVE ARABIDOPSIS AP2/ERF 59* (*ORA59*), two genes in the JA signaling pathway, and suppresses the expression of resistance-related genes *PR1* and *PR2* involved in SA signaling pathways [75]. Moreover, the *TaNAC1* gene also responds to treatments with exogenous JA or ET. Exogenous MeJA application decreases the expression of *TaNAC1* 3 and 6 h after treatment, but *TaNAC1* increases and peaks at 12 h. Likewise, ET treatment induces expression of *TaNAC1* which also peaks at 12 h after treatment. These results indicate that TaNAC1 may regulate both JA and SA signaling cascades [75]. Two homologous NAC TFs in tomato, JA2 and JA2L, were shown to regulate stomatal movement induced by pathogen infection. JA2 expression can be activated by ABA and promotes stomatal closure by regulating the expression of an ABA biosynthetic gene, while another NAC protein JA2L, the expression of which can be activated by JA, promotes stomatal reopening, indicating that these closely related NAC proteins play opposite functions in the regulation of pathogen-induced stomatal closure and reopening through distinct mechanisms [100].

#### 4.1.3. Cross-Talk between NAC TFs and ABA

The plant hormone ABA plays vital roles in abiotic stress responses, particularly in regulating the responses to drought, salinity and cold stresses [135,136]. Some studies have also suggested that ABA is an important regulator of pathogen-induced stress response [123,137]. ABA is known as an elicitor that induces stomatal closure. Stomata are not only passive channels through which pathogens can enter a plant, but are also active in innate immune responses [99]. Recent studies have found that aquaporins can facilitate the entrance of hydrogen peroxide into guard cells to mediate stomatal closure triggered by ABA and pathogen [138].

Sun et al. analyzed the differential expression profiles of 30 selected *ONAC* genes in response to ABA by qRT-PCR and found that the expression levels of 16 *ONAC* genes were up-regulated in rice seedlings 3 h after ABA treatment [139]. The expression of *ONAC066* was strongly activated by exogenous ABA, and over-expression of *ONAC066* enhanced the resistance to blast disease in rice [62] but significantly suppressed the expression of ABA-related genes and remarkably reduced endogenous ABA levels when the plants were inoculated with rice blast. These results indicate that ONAC066 may be a positive regulator in rice pathogen resistance by inhibiting ABA signaling pathways [62]. In barley, the application of exogenous ABA increased the basic resistance to *Bgh* in wild-type plants, but not in *HvNAC6* RNAi plants, and the expression of two ABA biosynthesis genes, *HvNCED1* (*9-CIS-EPOXYCAROTENOID DIOXYGENASE*) and *HvNCED2*, were reduced in *HvNAC6* RNAi plants, confirming that ABA is a positive regulator of basal resistance depending on HvNAC6 [81]. Taken together, these data demonstrate that HvNAC6 effectively maintains basal resistance against *Bgh* through modulating of ABA-mediated defense responses.

### 4.2. NAC TFs are Involved in ROS Signaling

ROS are not only important signal molecules, but are also toxic for plant cells. On the one hand, they play indispensable roles in many biological processes, such as plant growth, development and response to biotic and abiotic stimuli, but on the other hand, they can cause oxidative damage to DNA, proteins and membrane lipids [140,141,142]. The rapid microburst of ROS is a typical early defense response caused by pathogen infection [143,144,145], and localized production of H_2_O_2_ is one of the earliest and most detectable cytological defense responses when various fungal pathogens penetrate the plant cell wall [146].

Recently, Li et al. reported that the effector RxLR207 of the necrotrophic pathogen *Phytophthora capsici* can activate ROS-mediated cell death in *Nicotiana benthamiana*. RxLR207 is essential for virulence of *P. capsici*, targets and degrades the protein BINDING PARTNER OF ACD11, ARABIDOPSIS ACCELERATED CELL DEATH 11 (BPA1) and other BPA1-LiIKE PROTEINS (BPLs), and enhances ROS accumulation and cell death to promote pathogen infection [147]. However, necrotrophic pathogen differ significantly in infection strategy from biotrophic or hemibiotrophic pathogens, which try to reduce ROS production [148]. The *Puccinia* effector *Pst*GSRE1, which can be strongly induced early on during infection in wheat, targets TaLOL2, a ROS-associated TF that plays a positive role in biotic stress resistance, and prevents its nuclear localization. These actions of *Pst*GSRE1 suppress ROS-mediated cell death and compromise host immunity. In *PstGSRE1* RNAi plant line, the accumulation of H_2_O_2_ is significantly increased and the virulence of *Puccinia* is reduced, indicating that *Pst*GSRE1 can disrupt ROS-related plant defenses by disrupting localization of host immune response factors [148]. These data indicate that ROS homeostasis can be modulated during plant defense responses by different pathogens. 

NAC TFs have been proven to regulate ROS metabolism and homeostasis during the stress response. In rice, SNAC3 can enhance heat and drought tolerance by modulating ROS homeostasis [149,150]. In Arabidopsis, NAC WITH TRANSMEMBRANE MOTIF 1-LIKE 4 (NTL4) can directly bind to the promoter of *ARABIDOPSIS THALIANA RESPIRATORY BURST OXIDASE HOMOLOG* (*AtRBOH*) to trigger the generation of ROS under drought and high temperature, leading to leaf senescence [151]. ANAC013 mediates mitochondrial retrograde regulation by inducing expression of *MITOCHONDRIAL DYSFUNCTION STIMULON* (*MDS*), which significantly influences ROS production [150,152]. ANA0C17 can regulate the expression of *ALTERNATIVE OXIDASE1a* (*AOX1a*), which is a key player in mitochondrial ROS scavenging [153]. Furthermore, ANAC013 and ANAC017 can interact directly with RADICAL-INDUCED CELL DEATH1 (RCD1), which is also targeted by the effector HaRxL106 from *Hyaloperonospora arabidopsidis* [150,154], indicating that NAC proteins might play roles in ROS-associated pathogen defense signaling.

A rice orthologue of HvSNAC1, OsSNAC1, regulates ROS homeostasis through interacting with OsSRO1 (SIMILAR TO RCD (REGULATED CELL DEATH) ONE1) [83]. *OsSRO1c* is a SNAC1-targeted gene, which modulates stomatal closure and oxidative stress tolerance by regulating hydrogen peroxide [155]. OsNAC60 was reported to positively regulate rice disease resistance and was the target of a microRNA, miR164a [64]. Transient expression of *OsNAC60* in *N. benthamiana* induces ROS production, but miR164a does not induce ROS generation. Furthermore, ROS production was significantly reduced when OsNAC60 and miR164a were co-expressed, suggesting that miR164a negatively regulates the OsNAC60-mediated ROS production [64]. Together, these are numerous examples of the involvement of NAC TFs in plant disease resistance through regulating ROS production and its homeostasis.

## 5. Conclusions and Prospects

**Figure 3:**
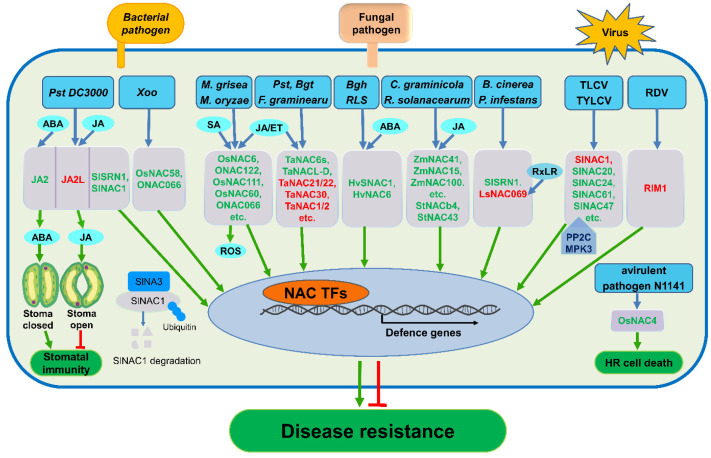
Overview of NACs signaling pathways during pathogen infection in main crops. NAC TFs participate in crop-pathogen interactions. NAC TFs can be induced upon infection with different pathogens, including bacteria, fungi and viruses, or be induced by treatment with exogenous defense signaling molecules, such as salicylic acid (SA), jasmonate (JA), ethylene (ET) and abscisic acid (ABA). NAC TFs cross-talk with phytohormones and other signals such as reactive oxygen species (ROS) to regulate downstream genes, stomatal immunity and the hypersensitive response (HR) to endow resistance against pathogens as positive regulators (green), or induce susceptibility to pathogens as negative regulators (red). Interactions between NAC TFs and other proteins are also shown. SlNAC1 can be ubiquitinated by the ubiquitin ligase SINA3 during *Pst* DC3000 infection, and several NAC TFs can interact with PP2C (protein phosphatase 2C) and MPK3 (mitogen-activated protein kinase (3) after TYLCV infection in tomato. Blue arrows indicate that NAC TFs are induced by various pathogens, hormones and other signaling molecules, the green arrows indicate that NAC TFs positively regulate downstream genes, stomatal immunity and HR against pathogens, and the red arrows indicate that NAC TFs negatively regulate stoma immunity and disease resistance. *Pst* DC3000, *Pseudomonas syringae* pv *tomato* DC3000; Xoo, *Xanthomonas oryzae* pv. *oryzae*; *M. grisea*, *Magnaporthe grisea*; *M. oryzae*, *Magnaporthe oryzae*; *Pst*, *Puccinia striiformis f*. sp. *tritici*; *Bgt*, *Blumeria graminis f.* sp. *tritici*; *F. graminearum, Fusarium graminearum*; *Bgh*, *Blumeria graminis f*. sp. *hordei*; *RLS*, ramularia leaf spot; *C. graminicola*, *Colletotrichum graminicola*; *R. solanacearum*, *Ralstonia solanacearum*; *P. infestans*, *Phytophthora capsici*; *B. cinerea*, *Botrytis cinerea*; TLCV, Tomato leaf curl virus; TYLCV, Tomato yellow leaf curl virus; RDV, Rice dwarf virus.

Global food demand is on a continuous rise at a time of increasing environmental deterioration, creating a situation where it is essential to increase the yield of common crops by improving their resistance to biotic and abiotic stresses. The NAC proteins comprise one of the largest TF families in plants and regulate a large number of cellular processes during both normal development and under times of stress. NAC TFs can be induced upon infection by different pathogens, including bacteria, fungi, and viruses, and interact with phytohormones, such as SA, ABA, JA, and ET, to either activate downstream defense genes, such as the *PRs* to endow resistance against pathogens as positive regulators, or to cause serious susceptibility to pathogens, as negative regulators (see Figure 3).

Since the discovery of NAC TFs over 20 years ago, the functional study of NAC TFs has attracted extensive attention. In recent years, great progress has been made in understanding how NAC TFs influence plant development and the responses to abiotic and biotic stresses in Arabidopsis and crops [13,156,157,158]. However, only a few studies on NAC TFs during the response to pathogens in main food crops have been reported, and there are still many unknowns to solve: What are the downstream targets and interaction partners of NAC TFs during pathogen infection? How do NAC TFs participate in defense regulatory networks? How can we use NAC TFs to improve crop tolerance to pathogens and their yields? Therefore, further research on NAC TFs should focus on: (1) Cloning and identifying new genes encoding NAC TFs from major crops by constructing new mutants and by bioinformatic analysis of public sequence databases; (2) Characterizing the structure and function of known and new NAC TFs of major crops in response to pathogens by genetics, biochemistry and molecular biology technologies; (3) Integrating NAC TF signaling into the networks of phytohormones and others signals such as ROS to elucidate the mechanisms by which NAC TFs improve resistance defense against pathogens by combining conventional molecular biology with multiple omics, such as transcriptomics, proteomics and metabolomics; and (4) Constructing engineered crops using CRISPR/Cas9 to knockout negative NAC TFs or knockin positive NAC TFs to improve crop resistance against pathogens and further increase quality and yield. CRISPR/Cas9 technology has become a mature, cutting-edge biotechnological tool for crop improvement that promises to accelerate the breeding of food crops [159,160]. All these in-depth studies of NAC TFs will increase our ability to improve stress resistance in crops to achieve agricultural sustainability for a growing world population.

## Data Availability

Not applicable.

## Supplementary Materials

The following are available online at https://www.mdpi.com/1422-0067/22/1/81/s1, Table S1: NAC protein sequences.

## Abbreviations

TF	Transcription factor
SA	Salicylic acid
JA	Jasmonic acid
MeJA	Methyl jasmonate
ET	Ethylene
ABA	Abscisic acid
ROS	Reactive oxygen species
PAMP	Pathogen-associated molecular patterns
PTI	PAMP triggered immunity
ETI	Effector-triggered immunity
PRRs	Pattern recognition receptors
WRKY	WRKYGQK
CAMTA	CaM-binding transcription activator
ERF/AP2	Ethylene responsive factor/ apetala2
TAR	Transcriptional activation region
NLS	Nuclear localization signal
NES	Nuclear export signal
TRR	Transcriptional regulatory region
TMM	Transmembrane motifs
HR	Hypersensitive response
PR	Pathogenesis-related
SAR	Systemic acquired resistance
SAG	SA O-β-glucoside
ChIP	Chromatin immunoprecipitation
